# An SOCP Estimator for Hybrid RSS and AOA Target Localization in Sensor Networks

**DOI:** 10.3390/s21051731

**Published:** 2021-03-03

**Authors:** Marcelo Salgueiro Costa, Slavisa Tomic, Marko Beko

**Affiliations:** 1 Cognitive and People-Centric Computing Labs (COPELABS), Universidade Lusófona de Humanidades e Tecnologias, Campo Grande 376, 1749-024 Lisboa, Portugal; slavisa.tomic@ulusofona.pt (S.T.); marko.beko@tecnico.ulisboa.pt (M.B.); 2 Instituto de Telecomunicações, Instituto Superior Técnico, Universidade de Lisboa, 1049-001 Lisbon, Portugal

**Keywords:** wireless sensor networks, target localization, optimization, Received Signal Strength (RSS), Angle of Arrival (AOA)

## Abstract

This work addresses the problem of target localization in three-dimensional wireless sensor networks (WSNs). The proposed algorithm is based on a hybrid system that employs angle of arrival (AOA) and received signal strength (RSS) measurements, where the target’s transmit power is considered as an unknown parameter. Although both cases of a known and unknown target’s transmit power have been addressed in the literature, most of the existing approaches for unknown transmit power are either carried out recursively, or require a high computational cost. This results in an increased execution time of these algorithms, which we avoid in this work by proposing a single-iteration solution with moderate computational complexity. By exploiting the measurement models, a non-convex least squares (LS) estimator is derived first. Then, to tackle its nonconvexity, we resort to second-order cone programming (SOCP) relaxation techniques to transform the non-convex estimator into a convex one. Additionally, to make the estimator tighter, we exploit the angle between two vectors by using the definition of their inner product, which arises naturally from the derivation steps that are taken. The proposed method not only matches the performance of a computationally more complex state-of-the-art method, but it outperforms it for small *N*. This result is of a significant value in practice, since one desires to localize the target using the least number of anchor nodes as possible due to network costs.

## 1. Introduction

Recent increased interest in wireless sensor networks (WSNs) in many fields is due to their ease of implementation, ability to operate in harsh environments, and relatively low cost [1,2]. Mainly for these reasons, many researchers gave rise to various investigation projects [3,4,5,6]. However, in many applications, the data acquired by sensors are only relevant if they can be associated with physical locations of the sensors, which can also enable self-configuration and autonomous operation of WSNs. Thus, our aim is to determine the locations of low-cost and low-power sensors that are deployed over a certain area in order to sense the information of interest. Perhaps the easiest way of localization is to equip sensors with global positioning system (GPS) receivers. However, this would increase the implementation cost, size, and energy consumption of the sensors. Besides, the use of GPS is not feasible in indoor or dense urban areas, nor in canyons and forests. Hence, different approaches are often required.

Typically, sensors are divided into two types: (1) target nodes—sensors whose locations are unknown, and (2) anchor nodes—sensors whose locations are known and used as reference points to localize the target nodes. Here, it is assumed that both types of nodes are capable of transmission, reception, and data-processing in order to execute the localization process.

In general, localization systems are based on (in)direct distance/direction observations, which can be obtained through radio measurements, such as received signal strength (RSS) [7,8], angle of arrival (AOA) [9,10,11], and so forth. Localization based on these two radio measurements is very important for future technologies, since RSS is practically available on all devices, while AOA is expected to be broadly available in the fifth generation of networks, due to the use of massive antenna arrays. Recently, measurement integration, such as AOA and RSS, has gained popularity [12,13,14,15], and will be the main focus of this work. Even though these hybrid techniques can enhance localization accuracy, they come at a certain cost, since additional equipment might be necessary in order to acquire the desired radio measurements [1]. The main problem of radio measurements is the non-linearity of the measurement models, which aggravates the process of solving the localization problem. A non-linear, that is, non-convex problem may have several locally optimal solutions and saddle points, and it is generally difficult to identify whether a solution is global or not. Therefore, various sub-optimal estimators have been proposed recently—see, for instance, [16,17,18,19,20].

Many advances related to RSS-AOA-based localization have been made recently, in both non-cooperative [21,22,23,24,25,26,27,28] (where targets are allowed to communicate with anchors exclusively) and cooperative networks [22,29] (where targets can communicate with any sensor within their communication range). In [21], the authors employed an unbiased constant which takes advantage of the link quality through the path loss and angle noise variance to solve the non-cooperative problem by means of weighted linear least squares. In [22], the authors considered both cases where the target’s transmit power is known and unknown. For the non-cooperative case, a squared-range approach was employed in order to convert the problem into a generalized trust region sub-problem and solve it via bisection, whereas in the cooperative case, semi-definite relaxation was used to convexify the problem. The authors in [23] proposed a Cartesian to spherical coordinates conversion approach in order to linearize the measurement models. Then, based on a weighted least squares (WLS) criterion, where weights were designed in such a way that they favor nearby links, a solution to the problem was derived. More recently, another linear estimator similar to the previously described approach was proposed in [24], where a different weighing strategy was introduced. The authors in [24] first obtained an initial solution to the problem by solving a LS problem (with no weights), after which this solution was exploited to calculate an approximate error covariance (EC) used to weigh the impact of each link. The downside of the works presented in [21,22,23,24] is that they are derived based on the assumption that the noise power is low, which might not hold in practice. Hence, these methods could suffer a great deterioration in their performance in noisy environments. It is also worth mentioning that both [21] and [24] were designed for the case where the target’s transmit power is perfectly known, and their generalization to the case of unknown transmit power is not straightforward.

In [25], the authors addressed the problem of optimal sensor placement strategy for single static target localization using the hybrid RSS, AOA, and time of arrival measurements in two-dimensional sensor networks. The authors in [12] presented a novel survival scheme for life detection after huge disaster situations based on portable cell phone detection system in a two-dimensional space. They considered two simulation settings in which they showed that their scheme based on RSS-AOA measurements works well, as long as certain variables are calibrated correctly. The work in [15] studied a target localization problem in three-dimensional networks using coupled RSS and AOA observations with unknown transmit power. The authors in [15] first derived a set of pseudo-linear equations from distance and angle equations, which (under the condition that the noise power is low) they successfully linearized by employing the first-order Taylor series expansion, resulting in a WLS estimator. Another WLS estimator for an unknown transmit power and path loss exponent is presented in [26]. The authors employed quantized values of analogue RSS measurements to jointly estimate the location of the target and the two aforementioned channel components, by first setting the two parameters to fixed values and estimating the location, after which an update of the parameters was performed. In [27], the authors took two different approaches based on a range-based least squares (RLS) and a squared range-based least squares (SRLS) to derive two distinct estimators based on semidefinite programming (SDP) relaxation for both cases of known and unknown transmit power. Although the proposed SDP estimators work well even in noisy environments, their computation complexity is very high, and for the case of unknown transmit power, the authors actually solve two SDP problems, which results in an increased execution time of the algorithms. In [28], the authors started by deriving pseudo-linear equations for the RSS and AOA measurement models. Based on a least squares (LS) criterion, a novel objective function was derived, which gave rise to a non-convex problem. In order to approximate it into a convex problem, the authors employed a combination of SDP and second-order cone programming (SOCP) relaxations, for both known and unknown transmit power cases. In [30], the authors used polarized identity to transform AOA measurements into squared-norm form, after which they employed the first-order Taylor series approximation and SOCP relaxation technique to derive their localization estimator. They considered both cases of known and unknown transmit power cases, where a three-step procedure was established for the latter one: solving the SOCP problem for unknown transmit power, estimating the value of the transmit power based on the obtained localization solution, and using the estimated transmit power to enhance the localization problem by solving the proposed SOCP problem as if transmit power was known. Lastly, in [29], the authors generalized their idea from [23] to the case of cooperative networks.

The works in [12,15,21,22,23,24,26,27,28,30] considered angle measurement noise to be a Gaussian random variable. However, the von Mises distribution is a more appropriate distribution to model angular noise [31]. Moreover, although most of the described methods show good localization accuracy, their computation complexity is still burdensome and not very suitable for real-time applications. Therefore, it will be shown here that the performance of the state-of-the-art method in terms of localization accuracy can be matched (or even surpassed) by a lighter estimator in terms of computational complexity. More precisely, in this work, we take a different approach to efficiently solve the localization problem in three-dimensional non-cooperative networks, where the target’s transmit power is not known. Unlike some of the existing methods that simply disregard the measurement noise from the derivation process, this work considers it as a residual error and tries to minimize it instead. To this extent, we derive the new estimator by applying SOCP relaxations, which arise naturally from simple manipulations of the measurement models. The proposed estimator outperforms the existing ones in terms of localization accuracy, especially in the case where anchor nodes are scarce. Hence, this result can be seen as a new lower bound on the achievable performance on the localization accuracy.

This paper is organized as follows. Section 2 introduces the measurement models and formalizes the localization problem. Section 3 provides a detailed derivation procedure of the proposed solution. Section 4 and Section 5 present and validate the performance of the proposed solution in terms of computational complexity and localization accuracy, respectively. Finally, Section 6 summarizes the main findings of this work.

## 2. Problem Formulation

**Figure 1:**
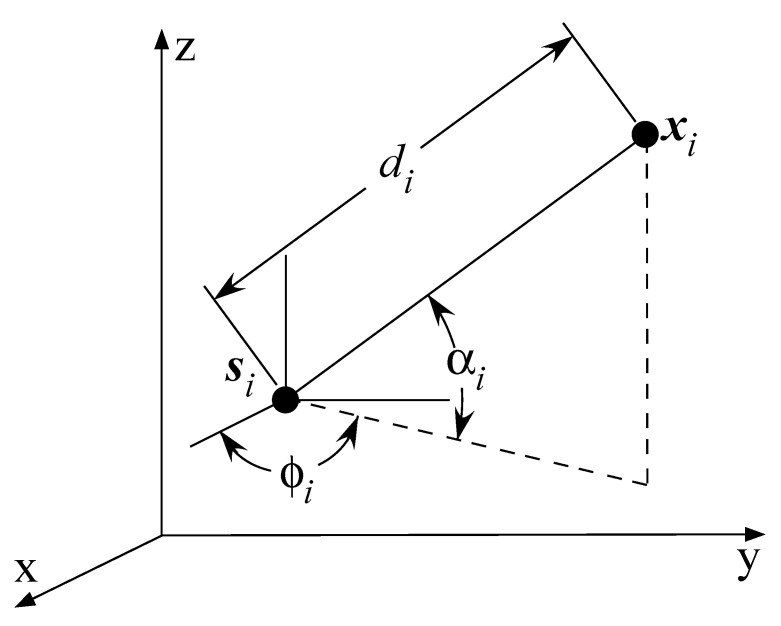
Illustration of a target and anchor in three-dimensional space, and the distance and bearing information between them.

Let us consider a 3D, non-cooperative WSN, where a single target node, $x\in R^{3}$, is located at a time by the help of a set of anchor nodes whose known locations are denoted by $s_{i}\in R^{3}$ for i=1,...,N. Figure 1 illustrates the considered localization scenario. The coordinates of the *i*-th anchor and the target are denoted by $s_{i}={\left(s_{ix},s_{iy},s_{iz}\right)}^{T}$ and $x={\left(x_{x},x_{y},x_{z}\right)}^{T}$, respectively, while $d_{i}$, $\phi_{i}$, $\alpha_{i}$ represent the true values of distance, azimuth, and elevation angles between the *i*-th anchor and the target, respectively. The goal of this work is to determine the unknown location of the target by resorting to a set of (indirect) distance and angle observations, together with the reference anchor’s location. These data are usually extracted from two different sensing modalities (e.g., distance is estimated in the time/frequency domain, while angle is estimated in the spatial/phase domain), which is why a common assumption in the existing literature is that the two measurement noises are uncorrelated [32,33], which was confirmed experimentally in [34].

According to [35], the path-loss $L_{i}=10\log_{10}\frac{P_{T}}{P_{i}}$, defined between the target and the *i*-th anchor, can replace the RSS model, where $P_{T}$ and $P_{i}$ represent the target’s transmit power and the RSS at the i-th anchor, respectively, as:

(1) $$L_{i}=L_{0}+10\gamma \log_{10}\frac{d_{i}}{d_{0}}+n_{i},$$

where $d_{i}=∥x-s_{i}∥$, $L_{0}$ denotes the path loss value at a short reference distance $d_{0}$ ($d_{i}\geq d_{0}$), γ is the path loss exponent (PLE) which represents the rate at which the signal strength decays with distance, and $n_{i}$ is the noise term modeled as a zero-mean Gaussian random variable with variance $\sigma_{n_{i}}^{2}$, i.e., $n_{i}∼N\left(0,\sigma_{n_{i}}^{2}\right)$. Notice that not knowing $P_{T}$ in the RSS model translates to now knowing $L_{0}$ in the path loss model.

According to Figure 1 and simple geometry, the azimuth and the elevation angles between the target and the *i*-th anchor (measured at the anchor) can be modeled, as

(2a) $$\begin{array}{c} \phi_{i}=\arctan\left(\frac{x_{y}-s_{iy}}{x_{x}-s_{ix}}\right)+m_{i}, \end{array}$$

(2b) $$\begin{array}{c} \alpha_{i}=\arctan\left(\frac{x_{z}-s_{iz}}{\left(x_{x}-s_{ix}\right)\cos\left(\phi_{i}\right)+\left(x_{y}-s_{iy}\right)\sin\left(\phi_{i}\right)}\right)+v_{i}, \end{array}$$

with $\phi_{i}\in \left(-\pi ,\pi \right)$, $\alpha_{i}=\left(-\frac{\pi }{2},\frac{\pi }{2}\right)$, where $m_{i}$ and $v_{i}$ in (2) represent the measurement error of the azimuth angle and the measurement error of the elevation angle. The two terms are modeled as von Mises random variables with zero-mean, whose concentration parameters are respectively denoted by $\kappa_{m_{i}},\kappa_{v_{i}}\in \left[0,\infty \right)$, that is, $m_{i}∼VM\left(0,\kappa_{m_{i}}\right)$ and $v_{i}∼VM\left(0,\kappa_{v_{i}}\right)$. This is different than in most of the existing approaches described in Section 1, where the angle measurement errors are assumed to be zero-mean Gaussian random variables. However, the Gaussian distribution is not generally appropriate for modelling AOA errors, because it has an infinite support instead of a periodic one (2π) across the angular domain [31]. Hence, it might not model the angle error reliably, and it is more appropriate to employ the von Mises distribution instead [31,36], which corresponds to a circular analogue counterpart of the Gaussian one. Nonetheless, one should note that there is a closed-form relationship between the mean direction and the concentration parameter of the von Mises distribution and the mean and variance of the Gaussian distribution [31,36]. With no loss of generality, and for the sake of notation simplicity, we assume that $\kappa_{m_{i}}=\kappa_{v_{i}}=\kappa_{i}$.

From (1), the conditional probability density function (PDF) of the path loss is given by

(3) $$f_{L_{i}}\left(L_{i}|x\right)\;=\;\frac{1}{\sqrt{2\pi \sigma_{n_{i}}^{2}}}\;\exp\;\left\{\;-\frac{{\left(L_{i}-L_{0}-\log_{10}\frac{d_{i}}{d_{0}}\right)}^{2}}{2\sigma_{n_{i}}^{2}}\;\right\}\;,$$

where exp{•} denotes the exponential function.

Similarly, from (2), the conditional PDF of the azimuth and elevation angles can be written as

(4a) $$\begin{array}{c} f_{\phi_{i}}\;\left(\;\phi_{i}|x\;\right)\;=\;\frac{1}{2\pi I_{0}\left(\kappa_{i}\right)}\exp\;\left\{\;\kappa_{i}\;\cos\left(\phi_{i}-{\hat{\phi }}_{i}\right)\;\right\}\;, \end{array}$$

(4b) $$\begin{array}{c} f_{\alpha_{i}}\;\left(\alpha_{i}|x\right)\;=\;\frac{1}{2\pi I_{0}\left(\kappa_{i}\right)}\exp\;\left\{\;\kappa_{i}\;\cos\left(\alpha_{i}-{\hat{\alpha }}_{i}\right)\;\right\}\;, \end{array}$$

where $I_{k}\left(•\right)$ is the modified Bessel function of first kind of order *k* [31,36], while ${\hat{\phi }}_{i}$ and ${\hat{\alpha }}_{i}$ denote the true azimuth and elevation angles between the target and the *i*-th anchor.

From (3) and (4), one can formulate a joint maximum likelihood (ML) estimator for the considered localization problem, by maximizing the conditional PDFs. Nevertheless, the ML estimator is non-convex, with no closed-form solution; thus, it will not be tackled directly here, but rather circumvented by deriving a convex estimator from the measurement models by the help of SOCP relaxation techniques.

## 3. The Proposed SOCP Estimator

This section describes the derivation process of the proposed SOCP estimator, for the case where the target’s transmit power is considered unknown.

First, by rearranging (1), one gets

(5) $$\begin{array}{cc} \; & ||x-s_{i}\left|\right|\epsilon_{i}=\lambda_{i}\eta , \end{array}$$

where $\epsilon_{i}=10^{\frac{n_{i}}{10\gamma }}$, $\lambda_{i}=10^{\frac{L_{i}}{10\gamma }}$, and $\eta =d_{0}10^{\frac{-L_{0}}{10\gamma }}$. Note that, since $L_{0}$ is assumed unknown, η is also unknown in (5). By slightly abusing strict mathematical formality, one can rewrite (5) as

(6) $$\epsilon_{i}=\frac{\lambda_{i}\eta }{∥x-s_{i}∥}.$$

Similarly, from (2), it follows that

(7a) $$\sin\left(\phi_{i}+m_{i}\right)\left(x_{x}-s_{ix}\right)=\cos\left(\phi_{i}+m_{i}\right)\left(x_{y}-s_{iy}\right),$$

(7b) $$\begin{array}{c} \begin{array}{cc} \; & \sin\left(\alpha_{i}+v_{i}\right)\left[\left(x_{x}-s_{ix}\right)\cos\phi_{i}+\left(x_{y}-s_{iy}\right)\sin\phi_{i}\right]\\ \; & =\cos\left(\alpha_{i}+v_{i}\right)\left(x_{z}-s_{iz}\right). \end{array} \end{array}$$

By assuming that the noise power is small and applying small-angle first-order approximations of the trigonometric function, that is, cosφ≈1 and sinφ≈φ, (7) can be rearranged as follows:

(8a) $$\begin{array}{c} \begin{array}{cc} \; & \rho_{i}\left(x-s_{i}\right)\approx m_{i}||x-s_{i}\left|\right|\cos\alpha_{i}, \end{array} \end{array}$$

(8b) $$\begin{array}{c} \begin{array}{cc} \; & \nu_{i}\left(x-s_{i}\right)\approx v_{i}||x-s_{i}\left|\right|, \end{array} \end{array}$$

where $\rho_{i}={\left[-\sin\phi_{i},\cos\phi_{i},0\right]}^{T}$ and $\nu_{i}={\left[-\sin\alpha_{i}\cos\phi_{i},-\sin\alpha_{i}\sin\phi_{i},\cos\alpha_{i}\right]}^{T}$. Similarly, as for the path loss case, (8) can be reformulated as

(9a) $$\begin{array}{cc} \; & m_{i}\approx \frac{\rho_{i}^{T}\left(x-s_{i}\right)}{||x-s_{i}\left|\right|\cos\alpha_{i}}, \end{array}$$

(9b) $$\begin{array}{cc} \; & v_{i}\approx \frac{\nu_{i}^{T}\left(x-s_{i}\right)}{||x-s_{i}\left|\right|}. \end{array}$$

Then, the target location can be estimated by solving the following problem, derived according to the LS principal applied to (6) and (9), as

(10) $$\begin{array}{cc} \;\underset{x,\eta }{\mathrm{minimize}}\;\sum_{i=1}^{N}{\left(\frac{\lambda_{i}\eta }{∥x-s_{i}∥}\right)}^{2} & +\sum_{i=1}^{N}{\left(\frac{\rho_{i}^{T}\left(x-s_{i}\right)}{∥x-s_{i}∥\cos\alpha_{i}}\right)}^{2}\\ \; & +\sum_{i=1}^{N}{\left(\frac{\nu_{i}^{T}\left(x-s_{i}\right)}{∥x-s_{i}∥}\right)}^{2}. \end{array}$$

The problem in (10) is non-convex, but it can be transformed into an SOCP as follows. First, introduce epigraph variables g, h, t, ($g,h,t\in R^{N}$) and define an auxiliary variable $y={∥x∥}^{2}$. Then, apply second-order cone relaxation of the form

$$\omega ={\left(\frac{\beta }{\tau }\right)}^{2}\;\Leftrightarrow \;∥\left[\begin{array}{c} 2\beta \\ \tau^{2}-\omega \end{array}\right]∥\;\leq \;\tau^{2}+\omega ,$$

and relax $y={∥x∥}^{2}$ into a convex constraint, that is, $y\geq {∥x∥}^{2}$.

**Figure 2:**
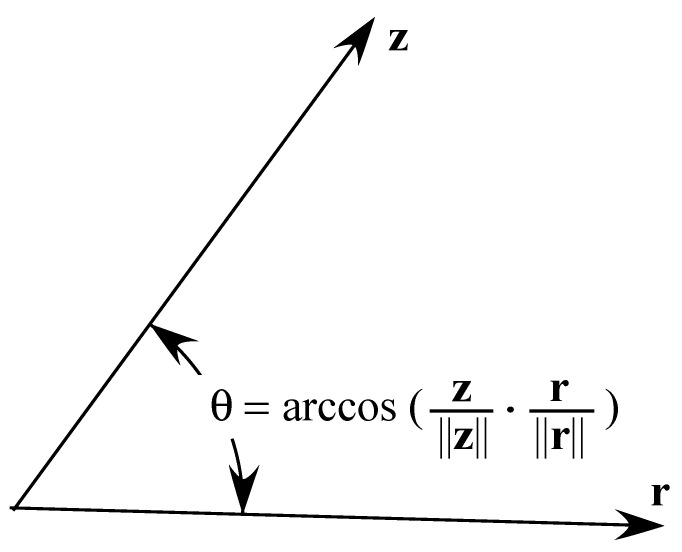
Geometric interpretation of the angle between two vectors using a dot product.

What has gone unnoticed so far in the literature is that ρ, ν and $\frac{\left(x-s_{i}\right)}{∥x-s_{i}∥}$ are all unitary vectors and, according to the definition of their dot products, we know that these actually represent a cosine of the angle between the respective pair of vectors, as illustrated in Figure 2. This means that the square of the cosines of the respective angles has to be between 0 and 1. Hence, this fact can help us tighten the restrictions of the proposed estimator, whose final form is given as the following SOCP problem.

(11a) $$\begin{array}{c} \underset{x,\eta ,g,h,t,y}{\mathrm{minimize}}\;\left(1_{N}^{T}g+1_{N}^{T}h+1_{N}^{T}t\right)\\ s.t\; \end{array}$$

(11b) $$\begin{array}{c} \begin{array}{c} ∥\left[\begin{array}{c} 2\lambda_{i}\eta \\ y-2s_{i}^{T}x+{∥s_{i}∥}^{2}-g_{i} \end{array}\right]∥\leq y-2s_{i}^{T}x+{∥s_{i}∥}^{2}+g_{i}, \end{array} \end{array}$$

(11c) $$\begin{array}{c} \begin{array}{cc} \; & ∥\left[\begin{array}{c} 2\frac{\rho_{i}^{T}\left(x-s_{i}\right)}{\cos\alpha_{i}}\\ \left(y-2s_{i}^{T}x+{∥s_{i}∥}^{2}\right)-h_{i} \end{array}\right]∥\leq \left(y-2s_{i}^{T}x+{∥s_{i}∥}^{2}\right)+h_{i}, \end{array} \end{array}$$

(11d) $$\begin{array}{c} \begin{array}{cc} \; & ∥\left[\begin{array}{c} 2\nu_{i}^{T}\left(x-s_{i}\right)\\ \left(y-2s_{i}^{T}x+{∥s_{i}∥}^{2}\right)-t_{i} \end{array}\right]∥\leq \left(y-2s_{i}^{T}x+{∥s_{i}∥}^{2}\right)+t_{i}, \end{array} \end{array}$$

(11e) $$\begin{array}{c} \begin{array}{cc} \; & \;0\leq h_{i}\leq \frac{1}{\cos^{2}\left(\alpha_{i}\right)},\;\;\;0\leq t_{i}\leq 1,\;\mathrm{for}\;i=1,...,N \end{array} \end{array}$$

(11f) $$\begin{array}{c} \begin{array}{cc} \; & ∥\left[\begin{array}{c} 2x\\ y-1 \end{array}\right]∥\leq y+1, \end{array} \end{array}$$

where $1_{N}$ denotes a column vector with all entries equal to 1 of size *N*, and the last constraint in (11) is an equivalent of the constraint $y\geq {∥x∥}^{2}$. Note that the restriction $h_{i}\leq \frac{1}{\cos^{2}\left(\alpha_{i}\right)}$ is troublesome when $\alpha_{i}\approx \pm \frac{\pi }{2}$, since we cannot divide by zero. Hence, we take this restriction into consideration only if $\alpha_{i}\in \left[-80,80\right]\times \frac{\pi }{180}$. We will refer to (11) as “SOCP” in the remaining text of this work.

## 4. Complexity Analysis

This section assesses the computation complexity of the proposed algorithm. The formula to calculate the worst-case computational complexity of an SDP/SOCP estimator is based on [37], and is given by

$$O\left(\sqrt{Q}\left(p\sum_{i=1}^{T_{sd}}l_{i}^{sd}+p^{2}\sum_{i=1}^{T_{sd}}l_{i}^{sd^{2}}+p^{2}\sum_{i=1}^{T_{soc}}l_{i}^{soc}+\sum_{i=1}^{T_{soc}}l_{i}^{soc^{2}}+p^{3}\right)\right),$$

where *Q* represents the iteration complexity of the algorithm, *p* represents the number of equality constraints, $T_{soc}$ and $T_{sd}$ are, respectively, the number of second-order cone and semidefinite constraints, and $l_{i}^{soc}$ and $l_{i}^{sd}$ represent, respectively, the dimension of the i-th second-order cone and the i-th semidefinite.

Table 1 summarizes the complexity analysis for the considered methods. It is worth mentioning that the estimators in [24,27] are composed of two iterations, and therefore, their complexities double, which also affects their execution time. Note that the ECWLS method is included here for comparison since it is considered here as the state-of-the-art method for the problem of interest in the case of known transmit power, but its generalization to the case where the transmit power is unknown is not straightforward.

Table 1 shows that the proposed estimator is the second least-complex method, which might have a positive impact on the lifetime of sensor batteries. Nevertheless, since integrated measurements are employed here, the localization process requires a very small number of anchors, and for these cases, the difference in the computational complexity is not dramatic. As we will see in the following section, the decreased computation complexity of the proposed estimator in comparison with the convex-based methods does not lead to a drop in localization accuracy. On the contrary, the new estimator not only matches the performance of the existing methods, but it outperforms them, especially for low *N*.

## 5. Numerical Results

This section assesses the performance of the considered methods in Table 1 in terms of localization accuracy summarized through Monte Carlo (MC) simulations. The simulations disclose the results for *N* randomly positioned anchors and one target within a three-dimensional space with an edge length of B=25 m. The received path loss at a reference distance $d_{0}=1$ m is set to $L_{0}=40$ dB, and the PLE is fixed to γ=2.5. However, it is not realistic that the value of the PLE is perfectly known in practice, since PLE can differ from link to link and it can vary in time. Hence, to account for a more realistic case, we define the true value of the PLE for each link, $\gamma_{i}$, as a uniform random variable on the interval [2.2, 2.8], that is, $\gamma_{i}∼U\left[2.2,2.8\right],i=1,...,N$ in each MC run. The root mean square error (RMSE), $\mathrm{RMSE}=\sqrt{\sum_{i=1}^{Mc}\frac{∥x_{i}-x_{i}^{\left(est\right)}{∥}^{2}}{Mc}}$ (m) and cumulative distribution function (CDF) of the localization error (LE), defined as ${\mathrm{LE}}_{i}=∥x_{i}-x_{i}^{\left(est\right)}∥$ (m), are used as performance metrics, where $x_{i}$ and $x_{i}^{\left(est\right)}$ are the true target location and the estimated target location in the *i*-th MC run, respectively.

The localization performance of the proposed method is compared with the ones summarized in Table 1. Moreover, we include the results for the Cramer-Rao lower bond (CRLB) as the theoretical bound on the achievable performance of any unbiased estimator [38].

**Figure 3:**
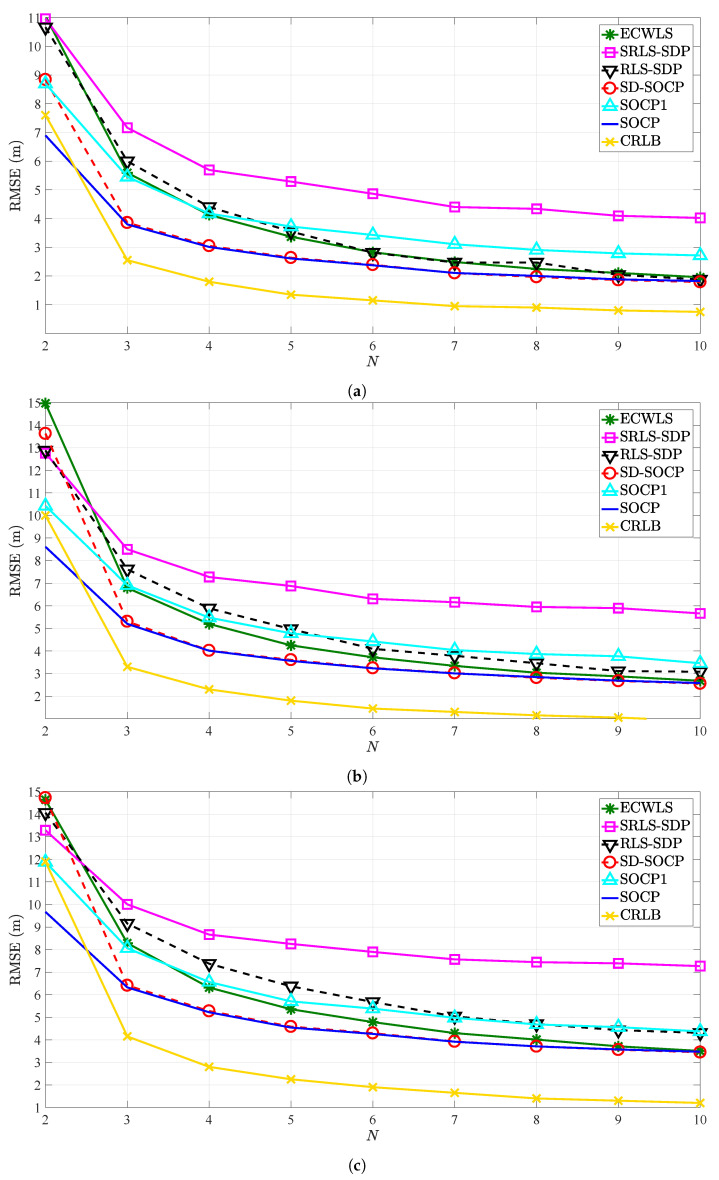
RMSE versus number of anchors, *N*, when γ=2.5, $\gamma_{i}∼U\left[2.2,2.8\right]$, B=25 m, Mc=5000. (**a**) $\sigma_{n_{i}}=4$ dB, $\kappa_{i}=45.8447$ (i.e., $\sigma_{m_{i}}=\sigma_{v_{i}}=6$ deg); (**b**) $\sigma_{n_{i}}=5$ dB, $\kappa_{i}=25.9034$ (i.e., $\sigma_{m_{i}}=\sigma_{v_{i}}=8$ deg); (**c**) $\sigma_{n_{i}}=6$ dB, $\kappa_{i}=16.6760$ (i.e., $\sigma_{m_{i}}=\sigma_{v_{i}}=10$ deg).

It is worth mentioning that the method in [24] was developed for the case of known $L_{0}$, and its generalization to the case where $L_{0}$ is not known is not straightforward. Hence, ECWLS is given the true value of $L_{0}$ in all simulations presented here. Figure 3 compares the RMSE (m) versus *N* for different values of noise powers. We point out that Figure 3 shows only the results of SOCP in [30], that is, “SOCP1”, for known transmit power. The main reason is that its performance for the case of unknown transmit power is even inferior; hence, for the sake of clarity, only the results of SOCP1 for known transmit power are included. One can observe from Figure 3 that the value of RMSE for all methods decreases with the increase of *N* for any choice of noise powers, as anticipated. Similarly, natural behavior is observed when the noise powers are increased, that is, the performance of all methods deteriorates with the increase of noise powers. The figure also shows that the proposed estimator matches the performance of SD-SOCP for N≥3 and outperforms all other considered ones for N=2, for any choice of noise powers. This result is of a significant value in practice, since one desires to localize the target using the least number of anchor nodes as possible due to network costs.

**Figure 4:**
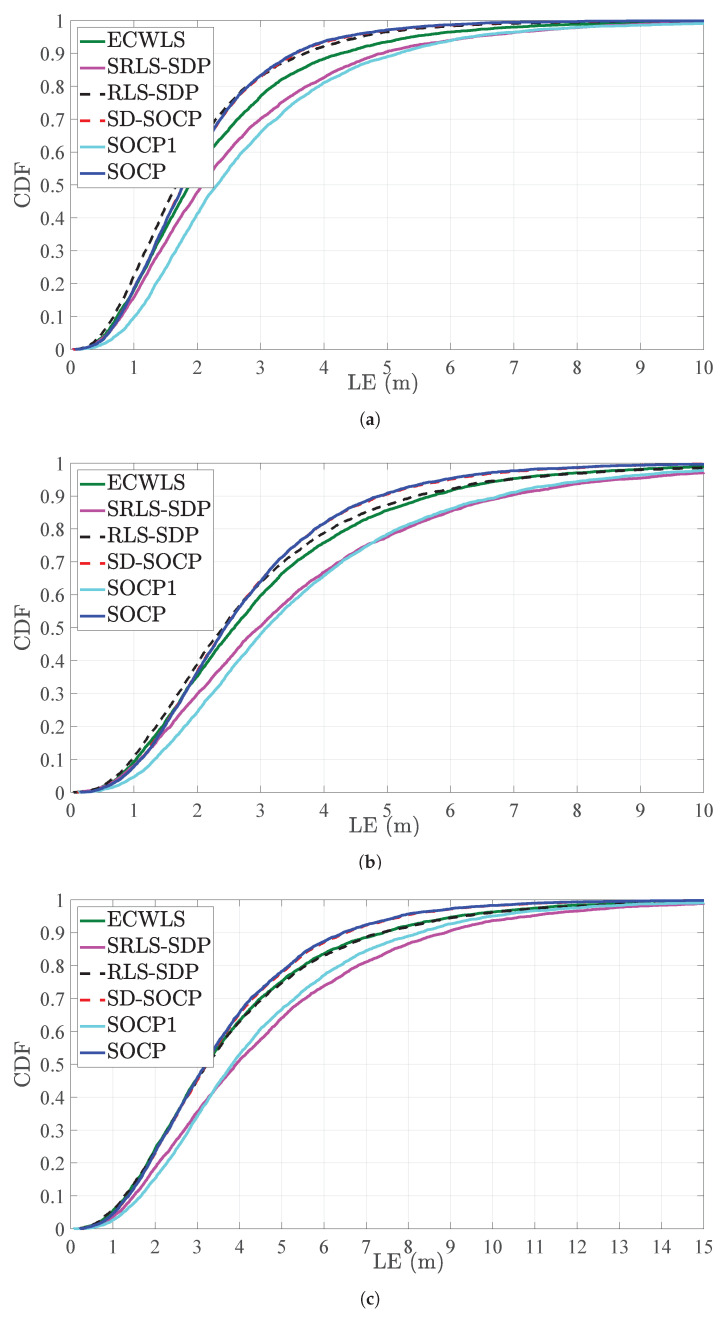
CDF versus LE (m), when N=6, γ=2.5, $\gamma_{i}∼U\left[2.2,2.8\right]$, B=25 m, Mc=5000. (**a**) $\sigma_{n_{i}}=4$ dB, $\kappa_{i}=45.8447$ (i.e., $\sigma_{m_{i}}=\sigma_{v_{i}}=6$ deg). (**b**) $\sigma_{n_{i}}=5$ dB, $\kappa_{i}=25.9034$ (i.e., $\sigma_{m_{i}}=\sigma_{v_{i}}=8$ deg). (**c**) $\sigma_{n_{i}}=6$ dB, $\kappa_{i}=16.6760$ (i.e., $\sigma_{m_{i}}=\sigma_{v_{i}}=10$ deg).

Figure 4 illustrates a CDF versus LE (m) comparison for different values of noise powers, for N=6. The figure exhibits that the proposed estimator achieves LE≤2 m (Figure 4a), LE≤3 m (Figure 4b), and LE≤5 m (Figure 4c) in almost 80% of the cases. This result is in concordance with results presented in Figure 3, where it was shown that the new estimator matches the performance of SD-SOCP for N=6.

## 6. Conclusions

In this paper, we have proposed an algorithm for hybrid RSS/AOA localization in three-dimensional, non-cooperative networks for unknown transmit power. This is a pertinent problem for forthcoming fifth-generation networks, where large bandwidths and highly directional communications are foreseen. We first derived a non-convex LS estimator based on the AOA and RSS measurement models. By resorting to convex relaxation techniques and exploiting the dot product definition, we were able to effortlessly transform the non-convex problem into a convex one, more precisely, an SOCP. The simulation results showed that the proposed approach outperformed the existing ones in general, even for the case where they were given perfect knowledge about the transmit power. The proposed estimator offers a new lower bound on the achievable RMSE performance, which was accomplished with significantly decreased computation complexity. Nevertheless, in our future work, we will focus on developing even less complex solutions to the problem, while maintaining the same, or even enhancing the localization accuracy.

## Figures and Tables

**Table 1:** Worst-case computational complexity of the considered methods.

Algorithm	Complexity
SOCP in (11)	$O(N^{3.5})$
ECWLS in [24]	2O(N)
RLS-SDP in [27]	$2O(N^{4.5})$
SRLS-SDP in [27]	$2O(N^{4.5})$
SD-SOCP in [28]	$O(N^{3.5})$
SOCP1 in [30]	$O(N^{3.5})$

## Data Availability

No new data were created or analyzed in this study. Data sharing is not applicable to this article.
